# The performance of bovine serum albumin filtration by using polyethersulfone-Tetronic 304 blend Ultrafiltration Membrane

**DOI:** 10.12688/f1000research.18740.2

**Published:** 2019-11-07

**Authors:** Fachrul Razi, Sri Mulyati, Nasrul Arahman

**Affiliations:** 1 Chemical Engineering Department, Universitas Syiah Kuala, Banda Aceh, Aceh, 23211, Indonesia; 2 Graduate School of Environmental Management, Universitas Syiah Kuala, Banda Aceh, Indonesia

**Keywords:** albumin, solute rejection, polyethersulfone, ultrafiltration

## Abstract

**Background**: Membrane technology has been widely applied for protein purification. In applications for protein separation, a membrane with stable filtration performance is necessary. In this work, two types of hollow fiber membranes with different characteristic were used to study the filtration profile of bovine serum albumin.

**Methods**: A single piece of hollow fiber module was used for ultrafiltration testing using UF0 and UFT304 membranes. Flux and rejection of BSA solution were collected based on a pressure-driven inside filtration model.

**Results**: Ultrafiltration experiments showed that the flux of UFT304 membrane was higher than that of UF0 membrane in all applied pressure condition. Solute rejection reaches 90 and 88% for ultrafiltration of BSA solution on the operating pressure of 0.5 atm using  UF0 and UFT304 membranes, respectively.

**Conclusion**: In general, UFT304 membranes has better ultrafiltration performance for BSA separation than UF0 membranes. The UFT304 membrane has a more stable flux for up to two hours of filtration.

## Introduction

Albumin proteins are one of the most important substances in the human body. Albumin maintains the balance of vascular fluid, forms new cell tissue, and functions acts as an antibody ^1^. Albumin can help to reduce the risk of stroke and coronary heart disease ^2^. Continuous experimentation is performed to establish the best method for isolation of protein from egg white to get the best quality albumin. The most popular technique is precipitation with Na _2_SO _4_ or (NH4) _2_SO _4_ at 25°C and pH 4–7. The protein-purifying method used in the precipitation technique can result in a large albumin yield, but the protein product contains the residue of precipitate materials, namely ammonium sulfate or sodium sulfate, which can potentially harmful ^3^. The low purity and loss of albumin during the purification process has led researchers to search for alternative methods to address these short-comings.

Rapid development in the field of biotechnology has allowed for the separation of proteins with safer and more efficient methods, namely purification using membrane technology (ultrafiltration, UF). This method has been reported to successfully achieve protein fractionation through UF with several advantages compared with the conventional separation using biology/chemistry methods ^4– 6^. The main advantage being generating a product with high purity level and low toxicity ^7^.

Despite these advantages, ultrafiltration technology also has some drawbacks. In many cases, protein molecules are easily absorbed on to the surface of hydrophobic membranes, such as polyethersulfone (PES), resulting in fouling. As a consequence, membrane flux decreases drastically along with the operation time. Modification of membrane surface traits is one solution to minimize the fouling of the membrane surface ^8, 9^.

In this research, the performance of two type hollow fiber membranes for BSA separation was investigated, one made of PES/ N-Methyl-2-pyrrolidone (NMP) and PES/NMP/Tetronic 304 system. Tetronic 304 is a non-ionic surfactant with two bonds of polyethylene oxide (PEO) and two bonds of polypropylene oxide (PPO) to ethylene diamine group. This surfactant potentially improves hydrophilic traits of the PES membrane due to its PEO group. This study aimed to investigate and establish the profile of BSA solution filtration using a standard PES membrane and a membrane modified with Tetronic 304 surfactant for permeability and rejection parameters.

## Methods

We used two types of hollow fibre membrane made of polyethersulfone (PES, E6020P: BASF Co. Ludwigshafen, Germany; FN. 218189-009)/N-Methylpyrrolidone (Merck Germany, 606-021-00-7) (labeled as UF0), and PES/NMP/Tetronic 304 (BASF, Germany) system (labeled as UFT304). UF0 membrane has inner and outer diameters of 699 and 776 µm, respectively. UFT304 membrane has an inner diameter of 705 µm and the outer diameter of 796 µm. The hydrophilicity characteristics based on water contact angle data are 74.0° and 62.0° for UF0 and UFT304 membrane, respectively. Protein model was bovine serum albumin (BSA) Cohn fraction V, pH 7 (WAKO Pure Chemical Industries, Japan; 017-23294).

### Characterization of hollow fibre membrane

Membrane characterization was performed to establish the morphology of all membrane types using scanning electron microscopy (SEM, Hitachi Co, S-800, Japan) with voltage acceleration at 20 kV, with a convergence angle of -30°, beam current x. 6.09 mm; y.5.79 mm, and diameter of the beam of 0.006 nm. Before the scanning electron microscopy (SEM) test, the membrane was soaked in liquid nitrogen for around five minutes, after the membrane turned rigid. This treatment was performed to prevent any change in the membrane structure when placing the sample on the SEM plate. The membrane was dried in freeze-drier overnight. One piece of the membrane was taken randomly and mounted on the sampling tubs (Hitachi, 10-004139-1, stubs Ø25 x 16mm x M4, 2 x 90°) wrapped with carbon tape, and then coated with osmium coater (Neoc-STB, Meiwafosis Co., Ltd., Japan) sputtering. The sample was placed on the SEM panel for the picture-capturing process at 100 and 3000 magnification for whole and enlarged cross-section, respectively. Captured images were processed using Irfan View v.3.45

### The designing of ultrafiltration module

The ultrafiltration module was designed using a single membrane module as previously described ^10^. Ultrafiltration module consisted of a peristaltic pump (Watson Marlow, SciQ 323), a circuit of hose for feed flow, two pressure gauges (WIKA 213.53), valve, and hollow fibre membrane installed in the circuit. It was also equipped with feed, permeate, and retentate container.

### Pure water, and albumin solution-separating process

The filtration of pure water and BSA solution was conducted by flowing the feed solution to the membrane using a peristaltic pump (Watson Marlow, SciQ 323). For pure water flux measurements, filtration was carried out at an operating pressure of 0.3; 0.5; 1.0; and 1.5 atm. Three pieces of the membrane were tested at every operating pressure by repeating three times. The permeate was collected every 10-minute filtration, and its weight was measured by an auto digital balance (Shimadzu, Japan; ATY224). The received data of permeate weight were then converted in a volume unit. The average of flux at each operating pressure is obtained from 9 times the filtration process. BSA solution filtration process were carried out for 120 minutes at a constant operating pressure of 0.5 atm. BSA solution was made at a concentration of 500 ppm at constant pH of 4.5. The permeate volume of BSA filtration was also collected every 10 minutes. The average permeability data were obtained from 3 measurements of each membrane.

Pure water flux and permeability of albumin solution were measured with Equation 1) and Equation 2) ^11^. The filtration process is carried out at several operating pressures, which are 0, 5; 1.00; and 1.5 Atm. 

$\mathrm{Flux}=\frac{V}{A.t}\;(1)$

$\mathrm{Permeability}=\frac{V}{A.t.P}\;(2)$

V is a permeate volume, A is the membrane surface area, t is filtration time, and P is operational pressure. BSA concentration in permeate and retentate was then analyzed using a spectrophotometer. Rejection capability of the membrane can be determined using the equations as follows ^11^:

$\mathrm{Rejection}=\frac{C_{F}-C_{p}}{C_{F}}\times 100\%\;(3)$

C _F_ and Cp are protein concentration in sample and permeate analyzed by a spectrophotometer UV-Vis (Shimadzu UV-1800) at a wavelength of 278.0.

## Result

### Membrane structure

**Figure 1.**
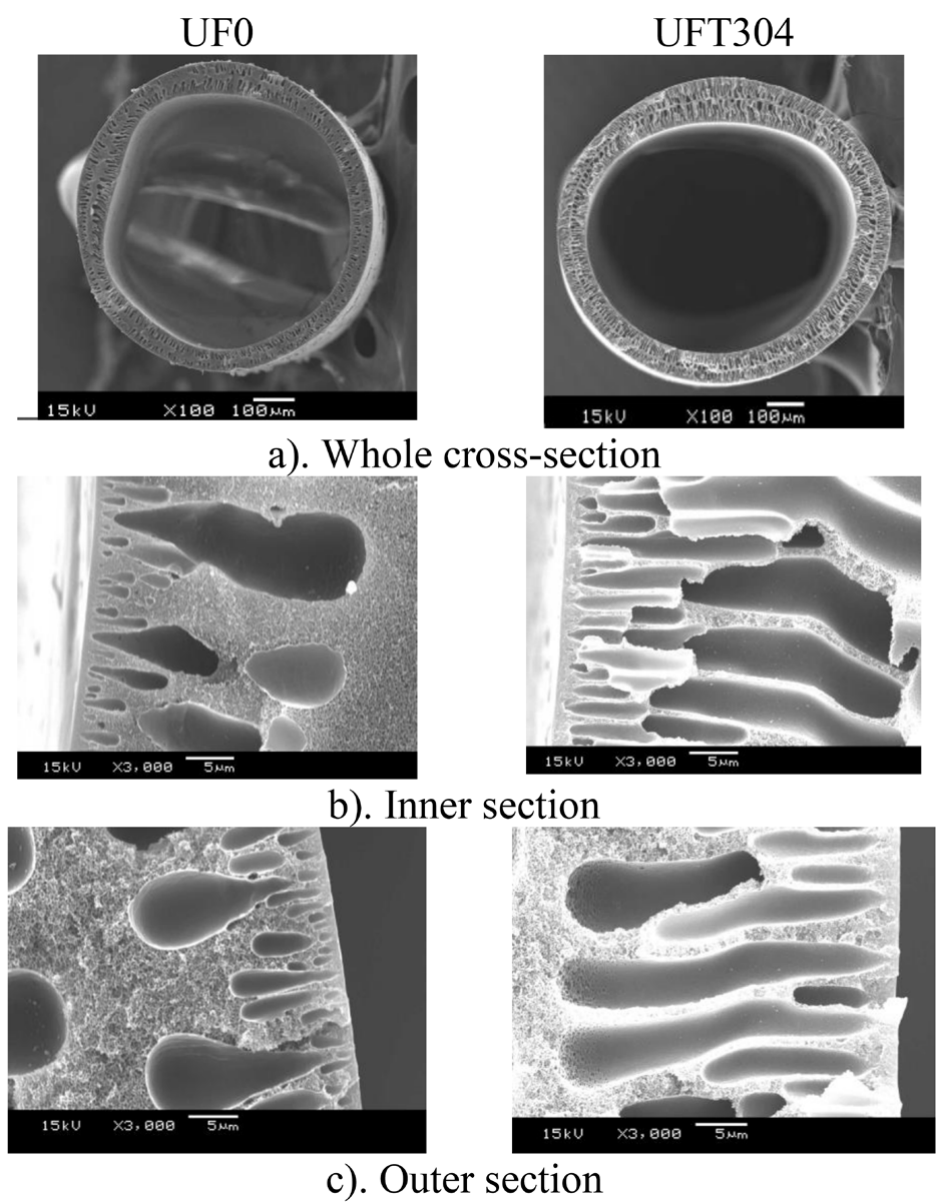
Membrane morphology observed by scanning electron microscopy (SEM).

SEM images of the two membrane are shown in Figure 1 (see underlying data ^12^). The morphological structure of membrane of the inner and outer surface were recorded with SEM at 3000x magnification as shown in Figure 1b and Figure 1c. Differences in pore structure and macrovoid pattern are clearly visible. The UFT304 membrane had larger pores with greater quantity of longer macrovoids. This likely effected ultrafiltration process of BSA protein as discussed below. Figure 1b and Figure 1c also shows a large sponge structure in the middle of UF0 membrane which is absent in the UFT304 membrane. The increase in number and size of macrovoid was caused by the addition of polymeric surfactant. During the solidification process of the membrane in the coagulation batch, part of the additive leached out of the polymer system. Therefore, a large macrovoid structure was formed.

### Flux and rejection of BSA

**Figure 2.**
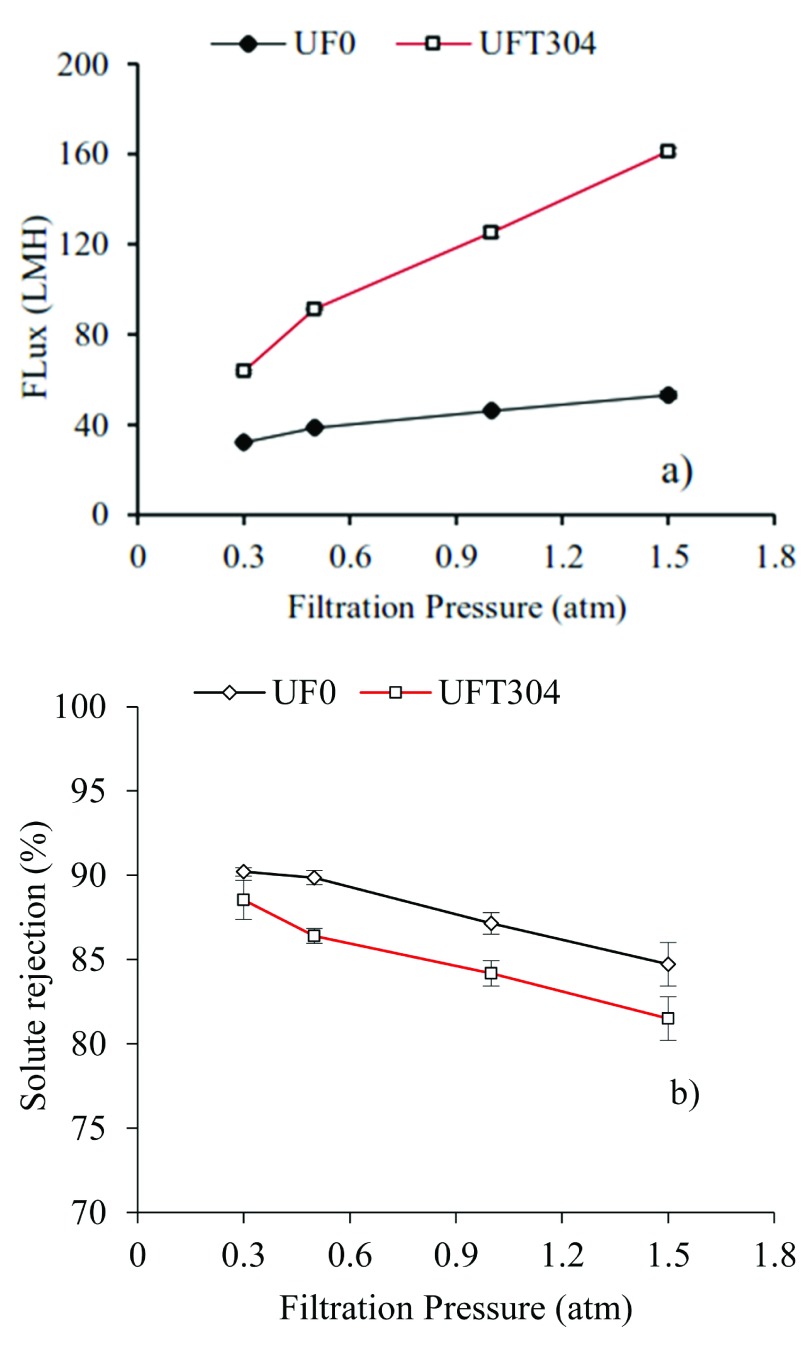
The ultrafiltration performance of membrane in term of flux ( **a**), and solute rejection ( **b**).

One important parameter for the membrane performance is its capability to filter solutions, referred to as flux or permeability. Another important parameter is the capability of membrane to remove components or particles from the solution, commonly known as solute rejection. In this study the ultrafiltration capability of the membrane was tested using a single membrane of hollow fibre (single module) designed with pressure driven inside (PDI) flow. The filtration profile of the albumin protein solution using UF0 and UFT304 membranes at various operational pressures is shown in Figure 2a (underlying data ^13^). The flux of UFT304 membrane at the beginning of this experiment with an operating pressure of 0.3 atm was 63.60 L/m ^2^.h. Flux increased significantly with the increase of the operating pressure which reached 161.00 L/m ^2^.hour at a pressure of 1.5 atm. The flux of UF0 membrane at the starting point (operating pressure 0.3 atm) was much lower, 32.0 L/m ^2^.h. At the highest operating pressure (1.5 atm), the flux of UF0 membrane was about 53.0 L/m ^2^.h.

The difference of flux between the two membranes is likely due to the differences in porosity. Tetronic 304 is a polymeric additive which acts as a pore-forming media. This additive is usually called the membrane-modifying agent (MMA). The increase of membrane pore size with blending MMA into the polymer solution is one of the easiest methods to improve membrane performance fabricated by the phase inversion method ^14^.

The rejection capability of albumin solution of the two membranes is shown in Figure 2b (underlying data ^15^). Albumin rejection by UF0 membrane >90% was achieved at 0.5 atm. Increasing the operating pressure resulted more albumin particles slipping into the permeate section. For all of the tested operating pressures the rejection of albumin solution by the two membranes remained above 80%.

### Filtration stability

**Figure 3.**
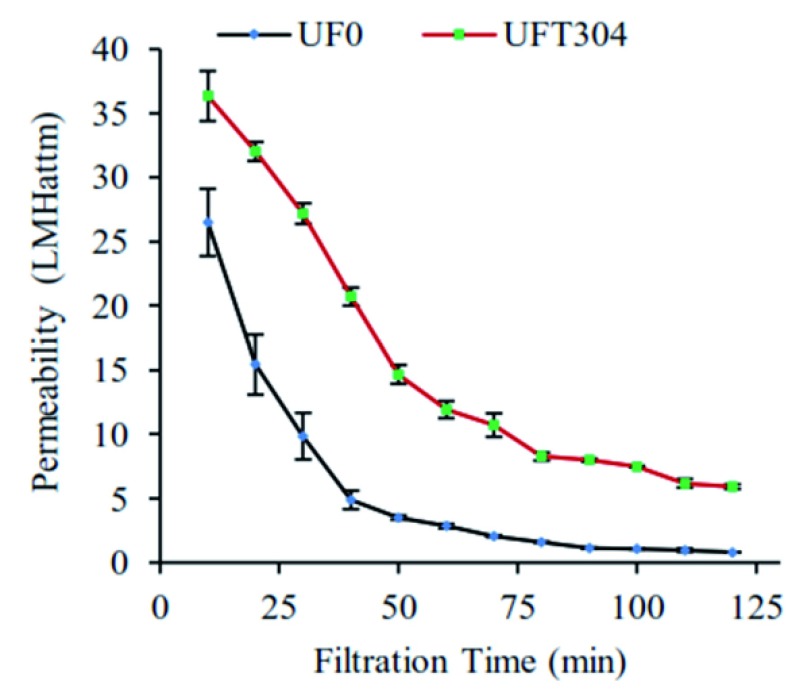
Filtration stability of UF0 and UFT304 membrane.

Filtration stability is the capability of a membrane to filter solute per time unit and operating pressure. This test corresponds to the durability of the membrane (lifetime). The result of stability test of the membrane on BSA filtration using UF0 and UFT304 membranes at 0.5 atm are shown in Figure 3 (underlying data ^16^).

The permeability of the UFT304 membrane at the initial operating pressure of 0.3 atm was 90.75 L/m ^2^.h.atm. Meanwhile, the permeability of UF0 membrane at 0.3 atm much lower, 14.38 L/m ^2^.h.atm. The performance of filtration in these two membrane types was closely related to the morphological structure of the membrane as shown in Figure 1. Compared with the UF0 membrane, the UFT304 membrane had a larger pore structure with a higher porosity. The structure of finger-like macrovoid was also longer, and there was no sponge structure in the middle of membrane section. This difference of pore structure likely resulted in the UFT304 membrane’s higher filtration capability.

Along with longer filtration time, the permeability of these two membrane types decreased due to the fouling in membrane pore surface. However, the permeability of UFT304 membrane was still at 5.60 L/m ^2^.h.atm after 120 minutes of filtration. In contrast the permeability of the UF0 membrane which was almost 0. The filtration profile of UFT304 membrane is more stable than UF0 membrane. The stability of filtration for UFT304 membrane was closely related to the hydrophilicity property of this membrane. UFT304 membrane was more hydrophilic with water contact angle of 62.0°, compared with UF0 membrane with water contact angle of 74.0°. The hydrophilic membrane has the capability to hold the fouling better than hydrophobic membrane ^17^.

The capability to reduce fouling in this hydrophilic membrane is related to the interaction force between membrane surface and solution particle (protein). The content of polyethylene oxide (PEO) chain in the Tetronic additive becomes a block (steric repulsion) for protein molecules, preventing them reaching the membrane surface ^18^. Thus, the protein molecule unlikely to attach the membrane surface. Therefore, the fouling process can be minimized.

## Conclusion

The application of ultrafiltration membrane technology to separate albumin protein was conducted with two types of hollow fibre membranes, namely UF0 and UFT304. Based on the results of SEM analysis, UFT304 membrane has more finger shape structures than UF0 membrane. The results of the ultrafiltration test showed that the UFT304 membrane has better performance in terms of the stability of the flux. The rejection of the BSA solution was higher than 80% for both membranes.

## Data availability

### Underlying data

Figshare: Supplementary data 1. https://doi.org/10.6084/m9.figshare.8109335.v1 ^13^

This project contains the following underlying data:

1. Membrane flux.csv (Raw data for membrane flux underlying Figure 2a)

Figshare: Supplementary data 2. https://doi.org/10.6084/m9.figshare.8109332.v3 ^15^

This project contains the following underlying data:

2. Membrane Rejection.csv (Raw data for solute rejection underlying Figure 2b)

Figshare: Supplementary data 3. https://doi.org/10.6084/m9.figshare.8109338.v2 ^16^

This project contains the following underlying data:

3. Water permeability of BSA solution.csv (Raw data for BSA filtration profile underlying Figure 3)

Figshare: SEM images. https://doi.org/10.6084/m9.figshare.8215862.v2 ^12^

This project contains the following underlying data:

- SEM images.pdf (Raw SEM images)

Data are available under the terms of the Creative Commons Attribution 4.0 International license (CC-BY 4.0).
