# Supplementary material for: The performance of bovine serum albumin filtration by using polyethersulfone-Tetronic 304 blend Ultrafiltration Membrane
Source: F1000Res. 2019 Nov 7;8:953. Originally published 2019 Jun 24. [Version 2] doi: 10.12688/f1000research.18740.2 (PMC6871366; doi:10.12688/f1000research.18740.2)
Supplement: Peer review history

# Peer review: Reviewer response for version 2

**DOI:** 10.5256/f1000research.23358.r56299

**Published:** 2019-11-20

**Authors:** Lisendra Marbelia

**Affiliations:** 1 Department of Chemical Engineering, Gadjah Mada University, Yogyakarta, Indonesia

I confirm that I have read this submission and believe that I have an appropriate level of expertise to confirm that it is of an acceptable scientific standard.

# Peer review: Reviewer response for version 2

**DOI:** 10.5256/f1000research.23358.r56298

**Published:** 2019-11-18

**Authors:** Alagumalai Nagendran

**Affiliations:** 1 Polymeric Materials Research Lab, PG & Research Department of Chemistry, Alagappa Government Arts College, Karaikudi, India

Author responded to my comments.

I confirm that I have read this submission and believe that I have an appropriate level of expertise to confirm that it is of an acceptable scientific standard.

# Peer review: Reviewer response for version 1

**DOI:** 10.5256/f1000research.20527.r54407

**Published:** 2019-11-01

**Authors:** Alagumalai Nagendran

**Affiliations:** 1 Polymeric Materials Research Lab, PG & Research Department of Chemistry, Alagappa Government Arts College, Karaikudi, India

F Razi et al. reported the performance of BSA filtration by PES-Tetronic 304 UF membranes. Authors have reported enough characterization and comprehensive discussion. It may be indexed and authors should consider the following issues:

1. Commercial membranes used made it less attractive. If we synthesize, it can optimize the performance characteristics much better. For example: BSA Rejection reported in this study is 80%. If we fabricate, still higher rejection can be achieved
2.  The role of Tetronic 304 in the modified membrane may be clearly explained in discussion section. Is it ok for long term operation?

I confirm that I have read this submission and believe that I have an appropriate level of expertise to confirm that it is of an acceptable scientific standard, however I have significant reservations, as outlined above.

# Response

**Published:** 2019-11-02

**Authors:** Nasrul Arahman

**Affiliations:** Syiah Kuala Uiversity, Indonesia

1. Commercial membranes used made it less attractive. If we synthesize, it can optimize the performance characteristics much better. For example: BSA Rejection reported in this study is 80%. If we fabricate, still higher rejection can be achieved

Response: Thank You. The statement raised by the reviewer is correct that the synthesized membrane can optimize the characteristic of membrane and its performance on filtration

  

1.  The role of Tetronic 304 in the modified membrane may be clearly explained in discussion section. Is it ok for long term operation?

Response: thank you for the valuable comments. In this study we examined for 2 hours filtration. More examination for long term operation is planned in our future study.

# Peer review: Reviewer response for version 1

**DOI:** 10.5256/f1000research.20527.r51291

**Published:** 2019-08-06

**Authors:** Lisendra Marbelia

**Affiliations:** 1 Department of Chemical Engineering, Gadjah Mada University, Yogyakarta, Indonesia

This article tries to compare the performance of two membranes type for BSA filtration. The two membranes are PES based membranes, UF0 and UFT304. Both membranes are PES/NMP based membranes, in which UFT304 is modified with Tetronic 304. 

 From the characterization in this study, it was seen that UFT304 is more hydrophilic (lower contact angle) and also more porous (fingerlike structure in SEM). Because of these properties, filtration results showed that UFT 304 has higher flux (fig 2.a) and also more sustained (fig 3) for a longer BSA filtration test, but with a slightly lower solute rejection (fig 2.b). 

 These above-mentioned finding and conclusions are obtained, shown and well explained in this article.

 Some suggestions and revision which should be applied to improve the quality of the paper are as follows: 

1. In the abstract, results part...It is written that UFT304 is better since it has higher flux. But for the rejection it is only mentioned for the UF0 membranes. Better mention for the UFT304.
2. In the introduction, last paragraph about the Tetronic modified PES membranes... please add reference here. 
3. In the method, more detailed information should be added on how the data were recorded. First, for the filtration with several pressure, how long was the filtration? Is it 10 minutes? Please write it accordingly. Also, related to the filtration experiments, it is not mentioned about the replications (in supplementary, it is shown that there are data replications). The authors should add this information in the methodology. How many replications and how the average data was calculated. Furthermore, the authors should add error bar to the graphs (2a, 2b and 3).
4. In the result, when discussing flux and rejection of BSA, it is written that the differences between the two membranes is due to differences in pore size. The authors should differentiate between pore size and porosity. Also in the discussion, it is mentioned pore density...It would be more appropriate to refer to this as porosity (finger like structure seen on SEM), otherwise it might be confused with pore size and density on the surface which are not observed in this study.  

Typos:

- Introduction, last paragraph - NMP instead of MNP
- Method, first paragraph - N-Methyl-2-pyrrolidone instead of N-metilpirrolidon 

I confirm that I have read this submission and believe that I have an appropriate level of expertise to confirm that it is of an acceptable scientific standard, however I have significant reservations, as outlined above.

# Response

**Published:** 2019-11-02

**Authors:** Nasrul Arahman

**Affiliations:** Syiah Kuala Uiversity, Indonesia

Comment 1

In the abstract, results part...It is written that UFT304 is better since it has higher flux. But for the rejection it is only mentioned for the UF0 membranes. Better mention for the UFT304.

Response

Thank You for comments. We revise the sentence as follow: Solute rejection reaches 90 and 88% for ultrafiltration of BSA solution on the operating pressure of 0.5 atm using UF0 and UFT304 membranes,  respectively

Comment 2.

In the introduction, last paragraph about the Tetronic modified PES membranes... please add

reference here. 

Response

Thank You for your suggestion. We putted a reference in last paragraph of introduction about the Tetronic modified PES membranes

Comment 3.

In the method, more detailed information should be added on how the data were recorded.

first for the filtration with several pressure, how long was the filtration? Is it 10 minutes?  Please write it accordingly. Also, related to the filtration experiments, it is not mentioned  about the replications (in supplementary, it is shown that there are data replications). 

The authors should add this information in the methodology. 

How many replications and how the average data was calculated. 

Furthermore, the authors should add error bar to the graphs (2a, 2b and 3).

Response: Thank You for reviewer suggestion. We have added the information in methods part. We have revised the graphs in Fig. (2a, 2b, 3) by including error bar.

Comment 4.

In the result, when discussing flux and rejection of BSA, it is written that the differences 

between the two membranes is due to differences in pore size. The authors should differentiate between pore size and porosity.  Also in the discussion, 

it is mentioned pore density.

It would be more appropriate to refer to this as porosity (finger like structure seen on SEM), 

otherwise it might be confused with pore size and density on the surface which are not

observed in this study.  

Response: The point raised by the reviewer is correct. We have revised our explanation as porosity.

Comment 5.

Typos:

Introduction, last paragraph - NMP instead of MNP

Method, first paragraph - N-Methyl-2-pyrrolidone instead of N-metilpirrolidon 

Response: Thank You for the correction. We have revised the typo
